# The evaluation of changes in maxillofacial bones using cone beam tomography in acromegaly

**DOI:** 10.4317/medoral.25280

**Published:** 2022-06-19

**Authors:** Fatma Dogruel, Emin Murat Canger, Zeynep Burcin Gonen, Firdevs Asantogrol, Ali Şahin, Fahri Bayram

**Affiliations:** 1 ORCID ID: 0000-0002-4290-2737. MD, Assistant Professor in Internal Medicine, Department of Oral and Maxillofacial Surgery, Faculty of Dentistry, Erciyes University, Kayseri, Turkey; 2 ORCID ID: 0000-0002-0798-9355. Associate Professor. Department of Oral and Maxillofacial Radiology, Faculty of Dentistry, Erciyes University, Kayseri, Turkey; 3 ORCID ID: 0000-0003-2725-9330. Associate Professor. Genome and Stem Cell Center, Department of Oral and Maxillofacial Surgery, Faculty of Dentistry, Erciyes University, Kayseri, Turkey; 4 ORCID ID: 0000-0002-0625-1359. DDS, Assistant Professor, Department of Oral and Maxillofacial Radiology, Faculty of Dentistry, Gaziantep University, Kayseri, Turkey; 5 ORCID ID: 0000-0001-7231-2394. Medical Doctor. Department of Neurosurgery, Kayseri Training and Research Hospital, Kayseri, Turkey; 6 ORCID ID:0000-0002-9637-6744. Professor. Department of Endocrinology and Metabolism, Faculty of Medicine, Erciyes University, Kayseri, Turkey

## Abstract

**Background:**

The aim of the study was to evaluate the changes in craniofacial dimensions of newly diagnosed and untreated acromegaly patients, patients with non-functional pituitary adenoma and healthy individuals on Cone Beam Computed Tomography (CBCT).

**Material and Methods:**

50 newly diagnosed acromegaly patients who did not receive any treatment for acromegaly were included in the study (Group A). Twenty patients with nonfunctional pituitary adenoma (Group B) and 30 healthy individuals were included (Group C). Linear, angular and volumetric measurements were performed.

**Results:**

Mandibular length showed significant difference in acromegaly patients, and maxillar length statistically significant difference was found between the A-B and B-C (*p*> 0,05), no difference was found between the A-C (*p*<0,05). SNB and ANB angle was statistically different in all groups, while SNA angle was statistically different between group A-C and B-C. In volumetric measurements, a statistically significant difference was found between groups a-c and groups A-B (*p*< 0,05), no difference was found between groups B-C (*p*>0,05).

**Conclusions:**

CBCT measurements showed that mandibular volume and length were increased in the acromegaly group compared to the group B-C. Present study is the first research that compares acromegaly patients in respect to changes in maxillofacial dimensions.

** Key words:**Acromegaly, pituitary adenoma, maxillofacial bones, cone beam computed tomography.

## Introduction

Acromegaly is a rare disease caused by adenoma which secretes the growth hormone (GH) in the pituitary gland. Its incidence is known as 6-8 in a million per year. Its prevalence is between 40 and 60 per million (1). It is known that the period between the onset and the diagnosis of the disease may extend to 6-10 years. Acromegaly causes an increase in morbidity and mortality due to metabolic effects formed by excessive secretion of GH and mass effect due to compression of pituitary adenoma.

Community studies have shown that the most common finding in acromegalic patients is the acral growth (78-85%) and facial roughening (70%). Headache, macroglossia, increased sweating, arthralgia, skin thickening, snoring, fatigue and carpal tunnel syndrome are other common findings (2).

The most prominent change in dental structures is the excessive growth of the jaw and the consequent prognathism. In addition, the teeth protrusion, diastema, thickened lips, enlarged nose and tongue, condylar hyperplasia, unfitting prosthesis in edentulous patients are other problems which can be seen (3,4). Most patients have a pathognomonic phenotype together with craniofacial abnormality when diagnosed. Arthropathies, characterized by the functional limitation and pain are common complications in acromegaly patients. It is known that the joint cavity is enlarged in the shoulder and knee joints and osteophyte formation occurs.

There are many changes mentioned above. However, it is not known what kind of changes in which bone causes this change. In this study, it was aimed to reveal the changes in facial bones in patients with acromegaly by bone measurements. Therefore, two groups were included in the study as the control group. These were healthy individuals and there were patients who had tumors in their brains but did not secrete hormones.

In this study, it is aimed to evaluate the changes in craniofacial ratios of newly diagnosed acromegaly patients who have not received any treatment and patients with non-functional pituitary adenoma and healthy individuals on 3D cone beam computed tomography (CBCT) images.

## Material and Methods

The present study planned in a prospective manner and was approved by the Ethics Committee of Erciyes University and was conducted in accordance with the principles of the Declaration of Helsinki. A written informed consent was obtained from each participant before the study.

In present study, 50 acromegaly patients (Group A), who were diagnosed recently with IGF-1 (19 female, 31 male; mean age 416 years) after examinations were made by the same endocrinology specialist (FB) in Erciyes University Endocrinology and Metabolic Diseases Department and who had not been treated for acromegaly, were included. The diagnosis of acromegaly was confirmed by measuring GH response to the oral glucose tolerance test (OGTT) and IGF-1 levels. Serum IGF-1 levels were also higher than the age- and sex-related reference ranges in acromegalic patients. Also patients with nonfunctioning pituitary adenoma was confirmed by measuring GH response to the oral glucose tolerance test (OGTT) and IGF-1 levels. The serum IGF-1 levels in patients with acromegaly were median 799.55 ng / ml (min: 368-max: 2384) and nonfunctioning pituitary adenoma patients were median 122.45 ng/ml (min:55-max:224).

Moreover, the pituitary magnetic resonance imaging (MRI) was performed at the time of diagnosis and MRI was evaluated by the same radiologist.

Twenty patients (10 females, 10 males; 50.113.1 years) with nonfunctioning pituitary adenoma (Group B), and 30 healthy individuals (15 females, 15 males; 49.312.4) were included as the healthy group (Group C). While the CBCT images of the patients with acromegaly and non-functional pituitary adenoma were taken for anato-morphological evaluation, the images of the healthy group were selected from patients who applied to Erciyes University Faculty of Dentistry for CBCT imaging for various reasons, who had no history of systemic disease in their medical history and who had the same FOV (field of view) range with the other two groups. All cone-beam tomography images were obtained using the CBCT device (NewTom 5G, Verona, Italy) in the supine position. The scan time was 18 seconds, the exposure time was 3.6 seconds and the voxel size was 0.25 mm. The images were evaluated using the NNT (NewTom 5G Cone Beam 3D imaging, Verona, Italy) computer program with a Dell LCD monitor with 32-inch 1,280 × 1,024-pixel resolution. Measurements were made by two oral radiologists who blinded to the clinical information of the subjects.

CBCT images were transferred to a computer program for 3D analysis (SimPlant, Materialize Medical USA) to assess the changes in the craniofacial morphology of the patients. Mandibular length, maxillary length, angular maxillomandibular relationship and face height were evaluated. The measurements of Temporomandibular Joint (TMJ) were evaluated by selecting the section with the widest mandibular condyle in the axial section. Then, in the coronal plane, the width of the mandibular fossa, the width of the mandibular condyle, the length of the joint cavity and the depth of the mandibular fossa were evaluated. The results of the patients were compared with patients with nonfunctioning pituitary adenoma and healthy subjects. According to our research, there is no many studies about the jaw bone.

- Measurements

**Figure 1:**
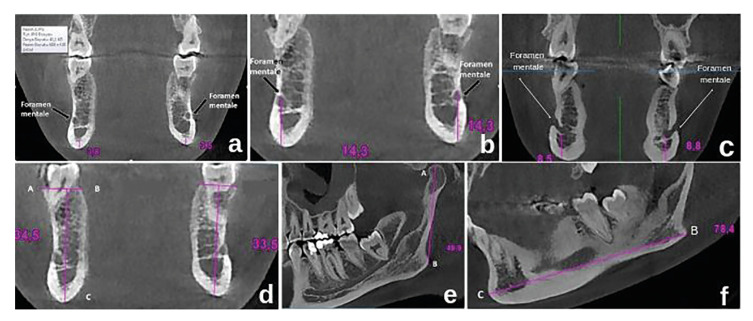
Reflecting measurements performed in mandible. a) Measurement of the cortical bone thickness bilaterally; b) Measurement of the distance between the upper border of foramen mentale and inferior border of mandible; c) Measurement of the distance between the lower border of foramen mentale and inferior border of mandible; d) Measurement of the alveoler crest height; e-f) Measurement of the length of mandible.

Cortical bone thickness: In coronal section CBCT images, the first section of the mental foramen was seen on both the right and left sides while moving from the anterior to posterior on the coronal plane. The mandibular cortical bone thickness was measured over these sections (Fig. 1).

The distance between the upper border of the mental foramen and the lower edge of the mandible: In the coronal section CBCT images, a line was drawn from the inferior border of the mandible to the superior border of the mental foramen; and the length of this line was measured (Fig. 1).

The Distance between Mental Foramen Lower Limit and Mandibular Lower Edge: In the coronal section CBCT images, a line was drawn from the inferior border of the mandible to the mental foramen’s superior border and the length of this line was measured (Fig. 1).

Alveolar Crest Height: In coronal section CBCT images, the distance between the lowest boundary of the mandible (C) and the line that connects the most protruding parts of the buccal (A) and lingual (B) of the alveolar crest in the molar teeth region was measured (Fig. 1).

Mandibular Length: In the sagittal cross-section CBCT images, a line extending from the most superior point of the mandible to the gonion (the most protruding point of the angulus mandibula) was drawn. A line was then drawn extending from the gonion to the pogonion (the most protruding spot of the lower jaw). The distances of these two lines were accepted as the length of the mandible (Fig. 1).

**Figure 2:**
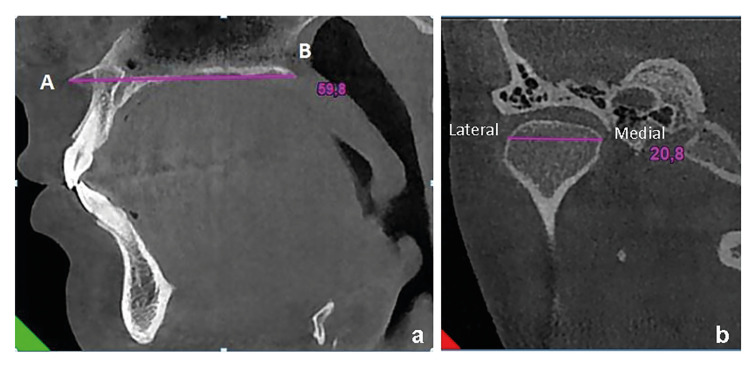
Reflecting the measurements performed in maxilla and mandibuler condyles. a) Measurement of the length of the maxilla; b) Measurement of the mediolateral width of the mandibuler condyles.

Maxilla Length: In the sagittal cross-section CBCT images, a section through the midline was found. The distance between the spinanasalis anterior (A) and the spinanasalisposterior was measured (Fig. 2).

Mandibular Condyle Mediolateral Width Measurement: The coronal section was selected as

the widest section in the mandibular condyle mediolateral direction and the length between the medial point and the lateral point was measured. This procedure was performed for both right and left, and the mean of both condyles was calculated (Fig. 2).

**Figure 3:**
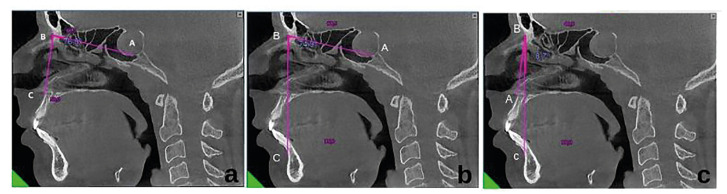
Orthodontic angle measurements. a) Measurement of the angle SNA (Sella-Nasion-A point) which indicates the position of the maxilla according to the skull base; b) Measurement of the angle SNB (Sella-Nasion-B point) which indicates the position of the mandible according to the skull base; c) Measurement of the angle ANB (A point-Nasion- B point) which indicates the position of the maxilla according to the mandible.

Sella Nasion A angle (SNA) measurement: In CBCT section crossing through the midline, a line was drawn from Sella point (A) to Nasion point (B). A line was then drawn from the Nasion point to point A (C), the deepest point of the pit between the spina nasalis anterior and prosthion. The angle between these two lines was measured. (Fig. 3)

Sella Nasion B angle (SNB) measurement: In the appropriate CBCT section, a line was drawn from Sella point (A) to Nasion point (B). A line was then drawn from Nasion point to point B (C), the deepest point of the pit between pogonion and infradentale. The angle between these two lines was measured (Fig. 3).

ANB angle measurement: A line was drawn from point A (A), the deepest point of the pit between the spina nasalis anterior and prosthion, to the point of Nasion (B). A line was drawn to point B (C), the deepest point of the pit between Nasion Pogonion and infrandentale. The angle between these two lines was measured (Fig. 3).

**Figure 4:**
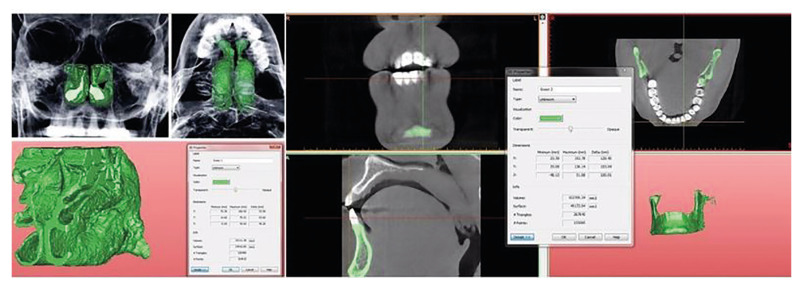
Volumetric measurements of nasal cavity and mandibuler body (including condyles). Measurements were carried automatically on SimPlant computer program after marking the region of interest manually.

Nasal Cavity and Mandibular Volume Measurement: CBCT images of the individuals were opened by using NNT program and was transferred to SimPlant ® Pro computer program. The images were adjusted to include the maximum pixel value in terms of the nasal cavity and mandible. Other adjustments were made manually. In the axial, coronal and sagittal planes, the connections of the nasal cavity and mandible with other anatomical structures were deleted. Afterwards, the nasal cavity (Fig. 4) and mandible volume (Fig. 4) were calculated as mm3 with the help of the software.

- Statistical Analysis

The obtained data were analyzed with Turcosa Cloud Statistical Software (Turcosa Cloud Software Co. Ltd. Kayseri, Turkey). The descriptive statistics were used to evaluate the distributions of the data. The compliance of the data for normal distribution was checked using the Shapiro-Wilk test. Levene test was performed since the data did not match the normal distribution in terms of the nasal cavity volume variable. The variances in terms of nasal cavity volume were distributed homogenously between the case groups; therefore, the Welch ANOVA test was used to compare between the groups. There was a significant difference between the groups; and Bonferroni correction t test was applied as a post-hoc test in order to understand in which groups the difference took place. *p*<0,05 was accepted statistically significant.

The post hoc power analysis was calculated by considering the effect sizes suggested by Cohen by estimating similar parameters in the literature. When the test group had at least 100 individuals for the regression method, the power of the test was calculated as 99,986% with 5% type 1 error (5).

## Results

Cortical bone thickness: While there was a statistically significant difference between the group A and group B (*p*<0,05), and group B and group C (*p*<0,05) in the right region, there was no difference between group A and group C (*p*>0,05). In the left region, there was a statistically significant difference between group A and group C (*p*<0,05), and group B and group C (*p*<0,05) while no difference was found between the group A and the group B (*p*>0,05) (Table 1).

The distance between the upper limit of the mental foramen and the lower edge of the mandibular: While there was no statistically significant difference between group A and group B (*p*>0,05) in the right region, there was a statistically significant difference between group A and group C (*p*<0,05) and between group B and group C (*p*<0,05). In the left region, a statistically significant difference was found among all three groups (*p*<0,05) (Table 1).

**Table 1:**
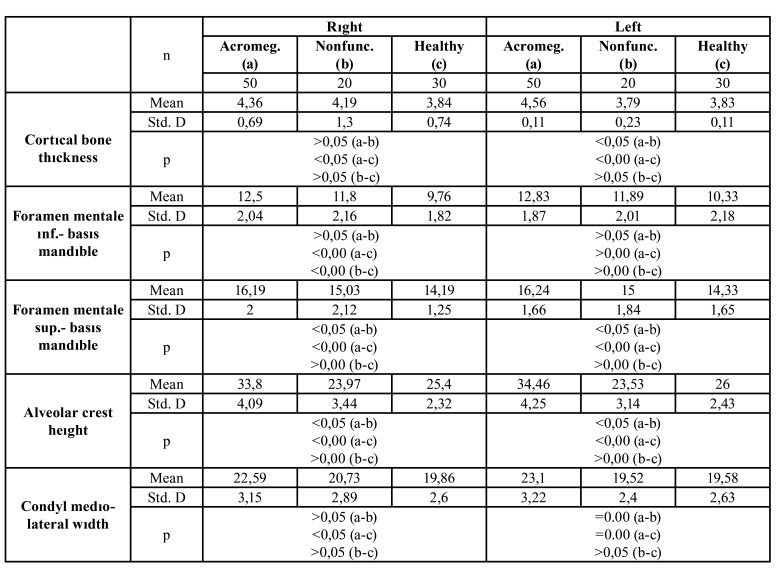
Distribution of bilateral linear measurements according to groups and regions.

The distance between the lower border of the mental foramen and the lower edge of the mandible: While there was no statistically significant difference between group A and group B (*p*>0,05) in both right and left regions, there was a significant difference between group A and group C (*p*<0,05) and between group B and group C (*p*<0,05) (Table 1).

Alveolar crest height: While there was no statistically significant difference between group A and group B (*p*>0,05) in both right and left regions, there was a significant difference between group A and group C (*p*<0,05), and between group B and group C (*p*<0,05) (Table 1).

Mandibular Length: Although there was a statistically significant difference between the group A - group C and the group A - group B (*p*<0,05), there was no significant difference between the group B and group C (Table 2).

Maxilla Length: While a statistically significant difference was found between group A and group B (*p*<0,05) and between group C and group B (*p*<0,05), no difference was found between

the group A and group C (*p*>0,05).

Mandibular Condyle Mediolateral Width Measurement: There was a statistically significant difference between group A and group B (*p*<0,05) and between group A and group C (*p*<0,05) while no difference was observed between the group B and group C (*p*>0,05) (Table 2).

SNA angle measurement: While there was no statistically significant difference between group A and group B (*p*>0,05), a difference was found between group A and group C (*p*<0,05) and between group B and group C (*p*<0,05) (Table 3).

SNB angle measurement: Statistically significant difference was found among all three groups (*p*<0,05). The highest measurement was in the acromegaly group (Table 3).

ANB angle measurement: While no statistically significant difference was found between group A and group B (*p*>0,05) and between group B and group C (*p*>0,05), there was a significant difference between group A and group C (*p*<0,05) (Table 3).

Nasal and mandibular volume measurement: In terms of nasal cavity volume; while a statistically significant difference was observed between group A and group B (*p*<0,05) and between group A and group C, no difference was found between group B and group C (*p*>0,05) (Table 4).

**Table 2:**
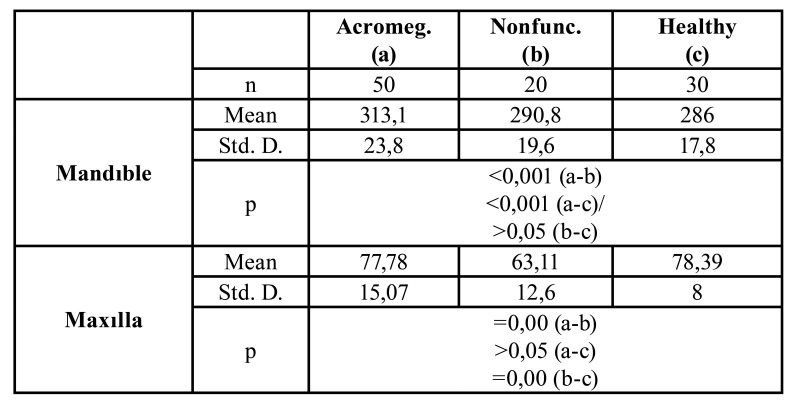
Distribution of jaw lenght measurements.

**Table 3:**
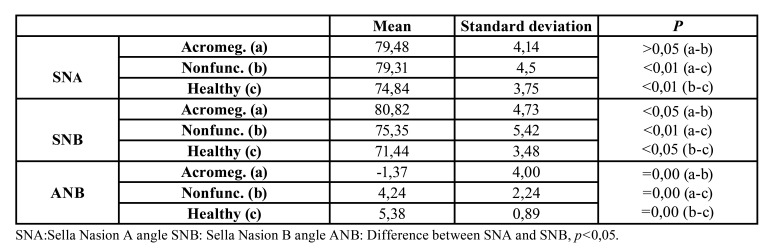
Orthodontic measurements and distribution between groups.

**Table 4:**
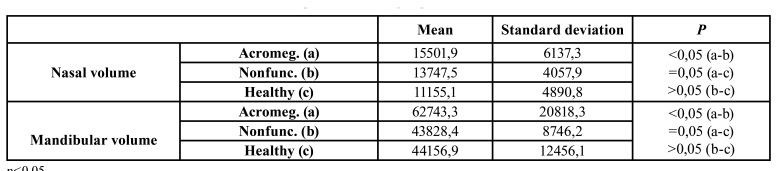
Nasal and mandibular volume results and comparison between groups.

In terms of mandible volume, while a statistically significant difference was found between group A and group C (*p*<0,05) and between group A and group B (*p*<0,05), no difference was found between group B and group C (*p*>0,05) (Table 4).

## Discussion

Acromegaly is an endocrine based disorder that occurs due to over secretion of the GH from pituitary gland. The most typical initial findings in patients with acromegaly are changes in the patient's face and jaw bones (2). The present study aimed to reveal the volumetric changes of facial bones with numerical data.

There are various studies designed to identify typical craniofacial alterations of patients with acromegaly (3,5-7). Present study was the first study includes patients which had been recently diagnosed (in one week prior to CBCT examination) and had never been received treatment. In addition, our study includes healthy individuals and non-functioning pituitary adenomas as the control group. Thus, the study has a different approach by investigating whether the strong effect on the change in the jaw bones is due to the IGF-1/GH axis.

Typical clinical findings of acromegaly occur over the years depending on levels of IGF-I, age, dimension of tumor, and delay in diagnosis. Therefore, its diagnose is delayed (4-8 years later than initiation). Average age of diagnosis is 40 and both genders effected equally (1,2). In present study, age of diagnosis was in concordance with literature and was 416 years. However, contrary to the literature male:female ratio was 1,6:1 (31 male, 19 female).

The course of the acromegaly continues with periosteal new bone production due to the excessive levels of GH and IGF-1 leads to growth of skeletal system. By the excessive secretion of GH and IGF-1after the closure of epiphyseal growth plate, a slow and nonproportional growth occurs in jaws and facial bones. This phenomenon can be responsible of late diagnosis.

Hormonal changes also induce the characteristic dental and craniofacial alterations (3). The most typical changes occur on facial bones. The most distinct is protrusion of glabella and increase in the vertical height of front face. Frontal bossing, paranasal sinus hypertrophy predominantly in frontal sinuses, widening of the nose, supra orbital and nuchal ridges happens. Characteristic diversity including mandibular prognatism, diastema and angle class III malocclusion arises. Reactivation of subcondylar growth centers, is responsible from this widening and prognatia of the mandible. In present study, mandibular length and condyle dimensions were statistically higher that other groups.

In general, although acromegaly is clinically diagnosed late, dental complaints of the patients like widening of the interdental spaces (diastema) forced them to apply to the dentist. Hence; the dentists are more prone to be first health worker who encounters acromegaly patients first (6,7). Except for orthodontic analysis studies, to our knowledge, no papers could be encountered in the literature evaluating all parameters of acromegaly patients adjusted in present study.

In general, the stimulation of GH-IGF-1 has adverse effects on the integrity of bone (8). Madeira *et al*. used distal radius by using high resolution peripheral quantitative tomography (HR-pOCT) and indicated an increase in the cortical bone thickness in eugonodal patients when compared with healthy individuals. Also results of present study indicated bilateral increase in

cortical bone thickness when compared with healthy individuals. This situation was linked to

the periosteal new bone apposition/deposition in acromegaly (7). It was reported that the position of foramen mentale wasn’t changed and kept constant despite increasing age and bone resorption (9). In present study, the distance between the foramen mentale and the base of mandible in both left and right sides was realized to be greater in group A and group B than in the group C. Neiva *et al*. (10) found the localization of the foramen mentale according to the lower border on mandibular cortex as 12 mm. (average of right and left), Aghtong *et al*. (11) found this distance 14 mm at right and 15 mm at left. Pele *et al*. (12) reviewed the literature and concluded that the mean distance from mandibular base to mental foramen ranged between 10 and 15 mm. In present study, in concordance with the literature, distance from mandibular base to mental foramen ranged between 9,76mm to 12,5 mm. Also, analysis of the facial dimensions of the patients was performed on CBCT images. Additionally, an asymmetry was reported about the localization according to the side.

Increases in the alveolar crest height, mandible length and mandible volume as well nasal cavity volume in acromegaly group is estimated to be explained with the bone growth notion seen in acromegaly. Differences in the nonfunctioning pituitary adenoma and healthy groups, the role of individual factors was estimated.

Excessive growth in mandibular condyles is one of the general radiographic and clinical findings of acromegaly (7) Beside its important role in the human growth and development, IGF-1 has also regulatory effect on the activity of growth plate. It organizes metabolism by increasing lipid, glycogen, and protein synthesis. It also has an impact by stimulating proliferation and differentiation of myeloblastic and osteoblastic tissues of bone and muscles (13). IGFs has an important factor for the neonatal and developing mandibular condyles, and may play role in mechanical transduction and osteoarthritis of TMJs. As IGF-II was found in relation with the etiology of some syndromes in which neonatal overgrowth is seen, a possible relationship between overgrowth of condyles and IGFs was also postulated (14,15). Increased levels of IGF-1 and IGF-1R was detected in cases of active condylar hyperplasia. In present study, it was found that mandibular condyles were wider in medio-lateral direction in acromegaly groups.

In orthodontics, two angulations are used to identify the position of maxilla and mandible according to skull base: SNA and SNB respectively. Additionally, ANB angulation is used to determine the position of maxilla and mandible in accordance with them. While an SNA angle which is higher than 82±2°indicates a more anterior position of maxilla, SNB angle higher than 80±2°indicates a protruded (prognathic) mandible. The standard value of angle ANB is 2±2°. While its positive value (> 00) is indicating forward position of maxilla according to mandible, negative value of it (<00), means maxilla is located behind of mandible. All orthodontic analysis studies on acromegaly were carried on lateral cephalometric radiographs. In terms of SNA angle Hermann *et al*. (3) indicated that no difference was found between acromegaly group and control group. In present study, value of SNB angle was higher in acromegaly group and indicates that mandible was located in front of maxilla. Naturally while ANB angle value was negative in acromegaly group, it was positive in control group. Kunzer and Farmand (16) and Dostolova *et al*. (4) stated that mandible was located minimally in front of maxilla. The limitation of Dostolova *et al*. (4) was that control group only consist of women. Similar to present study, Kunzer and Farmand (16) reported that mandibles were found to be longer than maxillas in acromegaly group.

In contrary with findings of present study, in their studies, acromegaly patients were compared with healthy control group, Pelttari *et al*. (17) found the ANB angle as 0,60 Karakis *et al*. (18) as 0,330 and Bavbek *et al*. (19) as 1,440. In both studies, mandible was found as positioned in the front according to maxilla. Kreitschmann- Andermahr *et al*. (20) conducted a questionnaire study on 145 patients and found mandibular enlargement in 35 patients and front positioning of mandible according to maxilla in 32 patients. Although having a different methodology, in point of SNA and SNB angles result of our study is in similar with the studies. But in terms of ANB angle, in contrary to the studies mentioned above. Additionally, statistically significant result was found between the acromegaly group and two other groups. In terms of gender, the distances between both the superior and inferior borders of foramen mentale and basis mandible, bilaterally alveolar crest height, left mandibular condyle width was found statistically significant in men. Also mandibular length was found statistically significant in men. In none of the studies above mentioned comparison of results according to the gender hadn’t been carried out.

According to the result of this study, bilaterally alveolar crest heights and mandibular lengths were found statistically significant according to the other groups. Also volumes of mandible and nasal cavity was found as significant in acromegaly group according to other groups.

Additionally, SNA and SNB angles were found as positive and ANB angle was found as negative in acromegaly group. Also in this study it was revealed that cortical bone thickness was high bilaterally in acromegaly group than control group and the distance between both superior and inferior borders of foramen mentale and basis mandible was high in group A and group B than the group C.

## Conclusions

The present study is important in respect to compare acromegaly patients, nonfunctional adenomic patients and healthy patients and is the first study comparing both three groups of patients. Dentists should be aware of that a patient with normal maxilla in the radiological and clinical examination but with an enlarged mandible in all 3 axes (Class III skeletal malocclusion) should be evaluated for acromegaly.
